# Supplementary material for: Confounds and overestimations in fake review detection: Experimentally controlling for product-ownership and data-origin
Source: PLoS One. 2022 Dec 7;17(12):e0277869. doi: 10.1371/journal.pone.0277869 (PMC9728858; doi:10.1371/journal.pone.0277869)
Supplement: S1 Table — (PDF) [file pone.0277869.s001.pdf]

Amazon review replacements

	Replacements		
**Matching**	**Brand**	**Random**	**Total**
Non-owners	123	35	158
Owners (truthful)	516	17	533
Owners (deceptive)	421	75	496
**Total**	1060	127	1187
