# Supplementary material for: Confounds and overestimations in fake review detection: Experimentally controlling for product-ownership and data-origin
Source: PLoS One. 2022 Dec 7;17(12):e0277869. doi: 10.1371/journal.pone.0277869 (PMC9728858; doi:10.1371/journal.pone.0277869)
Supplement: S2 Table — (PDF) [file pone.0277869.s002.pdf]

All features used in the classification experiments

Part of speech

POS_CC	POS_FW	POS_JJR	POS_NN	POS_PRP$	POS_RP	POS_VBN	POS_WDT
POS_CD	POS_IN	POS_JJS	POS_NNP	POS_RB	POS_VB	POS_VBP	
POS_DT	POS_JJ	POS_MD	POS_NNS	POS_RBR	POS_VBD	POS_VBZ	

LIWC

LIWC_Analytic	LIWC_adverb	LIWC_male	LIWC_achieve	LIWC_swear
LIWC_Clout	LIWC_conj	LIWC_cogproc	LIWC_power	LIWC_netspeak
LIWC_Authentic	LIWC_negate	LIWC_insight	LIWC_reward	LIWC_assent
LIWC_Tone	LIWC_verb	LIWC_cause	LIWC_risk	LIWC_nonflu
LIWC_WPS	LIWC_adj	LIWC_discrep	LIWC_focuspast	LIWC_AllPunc
LIWC_Sixltr	LIWC_compare	LIWC_tentat	LIWC_focuspresent	LIWC_Period
LIWC_Dic	LIWC_interrog	LIWC_certain	LIWC_focusfuture	LIWC_Comma
LIWC_function	LIWC_number	LIWC_differ	LIWC_relativ	LIWC_Colon
LIWC_pronoun	LIWC_quant	LIWC_percept	LIWC_motion	LIWC_SemiC
LIWC_ppron	LIWC_affect	LIWC_see	LIWC_space	LIWC_QMark
LIWC_i	LIWC_posemo	LIWC_hear	LIWC_time	LIWC_Exclam
LIWC_you	LIWC_negemo	LIWC_feel	LIWC_work	LIWC_Dash
LIWC_shehe	LIWC_anx	LIWC_bio	LIWC_leisure	LIWC_Quote
LIWC_they	LIWC_anger	LIWC_body	LIWC_home	LIWC_Apostro
LIWC_ipron	LIWC_sad	LIWC_health	LIWC_money	LIWC_Parenth
LIWC_article	LIWC_social	LIWC_ingest	LIWC_relig	LIWC_OtherP
LIWC_prep	LIWC_family	LIWC_drives	LIWC_death	
LIWC_auxverb	LIWC_friend	LIWC_affiliation	LIWC_informal	

Bigrams

BI_also_batteri	BI_everi_day	BI_like_phone	BI_phone_much	BI_samsung_galaxi
BI_android_phone	BI_front_camera	BI_look_good	BI_phone_price	BI_sd_card
BI_batteri_life	BI_good_phone	BI_much_better	BI_phone_realli	BI_smart_phone
BI_bought_phone	BI_good_price	BI_new_phone	BI_phone_screen	BI_sound_qualiti
BI_camera_good	BI_great_phone	BI_oper_system	BI_phone_work	BI_take_photo
BI_camera_phone	BI_intern_storag	BI_phone_batteri	BI_phone_would	BI_use_phone
BI_camera_qualiti	BI_iphon_7	BI_phone_camera	BI_pretti_good	BI_want_phone
BI_cheap_phone	BI_ive_ever	BI_phone_ever	BI_qualiti_phone	BI_work_well
BI_dont_know	BI_ive_phone	BI_phone_everyth	BI_realli_good	BI_would_recommend
BI_dont_want	BI_last_day	BI_phone_good	BI_recommend_phone	
BI_even_though	BI_last_long	BI_phone_ive		

Unigrams

UNI_1	UNI_buy	UNI_especi	UNI_high	UNI_mention	UNI_product	UNI_spec
UNI_10	UNI_call	UNI_etc	UNI_hit	UNI_might	UNI_purchas	UNI_specif
UNI_2	UNI_came	UNI_even	UNI_home	UNI_mine	UNI_put	UNI_speed
UNI_2020	UNI_camera	UNI_ever	UNI_honestli	UNI_mobil	UNI_qualiti	UNI_spend
UNI_3	UNI_cant	UNI_everi	UNI_hour	UNI_model	UNI_quickli	UNI_star
UNI_4	UNI_card	UNI_everyday	UNI_howev	UNI_money	UNI_quit	UNI_start
UNI_5	UNI_case	UNI_everyth	UNI_huawei	UNI_month	UNI_ram	UNI_still
UNI_6	UNI_caus	UNI_excel	UNI_huge	UNI_much	UNI_rang	UNI_storag
UNI_7	UNI_cellphon	UNI_except	UNI_id	UNI_multipl	UNI_rate	UNI_store
UNI_8	UNI_chang	UNI_expect	UNI_im	UNI_music	UNI_rather	UNI_super
UNI_abl	UNI_charg	UNI_expens	UNI_imag	UNI_must	UNI_read	UNI_support
UNI_absolut	UNI_cheap	UNI_experi	UNI_import	UNI_need	UNI_real	UNI_suppos
UNI_access	UNI_cheaper	UNI_extra	UNI_impress	UNI_never	UNI_realli	UNI_sure
UNI_actual	UNI_choic	UNI_extrem	UNI_includ	UNI_new	UNI_reason	UNI_system
UNI_addit	UNI_clear	UNI_fact	UNI_instal	UNI_next	UNI_recent	UNI_take
UNI_afford	UNI_color	UNI_fail	UNI_intern	UNI_nice	UNI_recommend	UNI_thank
UNI_ago	UNI_come	UNI_fall	UNI_internet	UNI_normal	UNI_record	UNI_that
UNI_allow	UNI_compani	UNI_far	UNI_iphon	UNI_noth	UNI_releas	UNI_thing
UNI_almost	UNI_compar	UNI_fast	UNI_isnt	UNI_notic	UNI_reliabl	UNI_think
UNI_alreadi	UNI_complet	UNI_faster	UNI_issu	UNI_offer	UNI_resist	UNI_though
UNI_also	UNI_condit	UNI_featur	UNI_ive	UNI_often	UNI_resolut	UNI_time
UNI_although	UNI_connect	UNI_feel	UNI_job	UNI_ok	UNI_respons	UNI_took
UNI_alway	UNI_consid	UNI_find	UNI_keep	UNI_okay	UNI_review	UNI_top
UNI_amaz	UNI_cool	UNI_fine	UNI_know	UNI_old	UNI_right	UNI_total

UNI_amount	UNI_cost	UNI_fingerprint	UNI_lack	UNI_one	UNI_run	UNI_touch
UNI_android	UNI_could	UNI_first	UNI_lag	UNI_open	UNI_samsung	UNI_tri
UNI_annoy	UNI_couldnt	UNI_fit	UNI_larg	UNI_oper	UNI_satisfi	UNI_turn
UNI_anoth	UNI_coupl	UNI_flagship	UNI_last	UNI_option	UNI_say	UNI_two
UNI_anyon	UNI_cover	UNI_found	UNI_least	UNI_os	UNI_scratch	UNI_updat
UNI_anyth	UNI_crash	UNI_freez	UNI_less	UNI_other	UNI_screen	UNI_upgrad
UNI_app	UNI_current	UNI_friend	UNI_let	UNI_overal	UNI_sd	UNI_usag
UNI_appl	UNI_custom	UNI_front	UNI_lg	UNI_own	UNI_second	UNI_use
UNI_applic	UNI_daili	UNI_full	UNI_life	UNI_pay	UNI_see	UNI_user
UNI_arent	UNI_damag	UNI_function	UNI_light	UNI_peopl	UNI_seem	UNI_valu
UNI_around	UNI_data	UNI_galaxi	UNI_like	UNI_perform	UNI_servic	UNI_version
UNI_ask	UNI_day	UNI_game	UNI_line	UNI_person	UNI_set	UNI_video
UNI_avail	UNI_deal	UNI_gave	UNI_littl	UNI_phone	UNI_sever	UNI_want
UNI_away	UNI_decent	UNI_gb	UNI_live	UNI_photo	UNI_short	UNI_wasnt
UNI_back	UNI_decid	UNI_gener	UNI_load	UNI_pick	UNI_show	UNI_watch
UNI_bad	UNI_definit	UNI_get	UNI_long	UNI_pictur	UNI_simpl	UNI_way
UNI_basic	UNI_design	UNI_give	UNI_longer	UNI_piec	UNI_simpli	UNI_week
UNI_bateri	UNI_devic	UNI_given	UNI_look	UNI_pixel	UNI_sinc	UNI_well
UNI_batteri	UNI_didnt	UNI_go	UNI_lose	UNI_play	UNI_singl	UNI_whole
UNI_beauti	UNI_differ	UNI_goe	UNI_lot	UNI_plu	UNI_size	UNI_within
UNI_believ	UNI_disappoint	UNI_good	UNI_love	UNI_pocket	UNI_slightli	UNI_without
UNI_besid	UNI_display	UNI_googl	UNI_low	UNI_point	UNI_slow	UNI_wont
UNI_best	UNI_doesnt	UNI_got	UNI_lower	UNI_poor	UNI_small	UNI_work
UNI_better	UNI_dont	UNI_great	UNI_made	UNI_power	UNI_smart	UNI_worth
UNI_big	UNI_download	UNI_half	UNI_main	UNI_present	UNI_smartphon	UNI_would
UNI_bigger	UNI_drop	UNI_hand	UNI_make	UNI_pretti	UNI_softwar	UNI_wouldnt
UNI_bit	UNI_due	UNI_handl	UNI_mani	UNI_previou	UNI_someon	UNI_wrong
UNI_bought	UNI_easi	UNI_happi	UNI_market	UNI_price	UNI_someth	UNI_x
UNI_brand	UNI_easili	UNI_hard	UNI_may	UNI_pro	UNI_sometim	UNI_xiaomi
UNI_bright	UNI_els	UNI_hardwar	UNI_mayb	UNI_probabl	UNI_soon	UNI_year
UNI_bring	UNI_end	UNI_heavi	UNI_mean	UNI_problem	UNI_sound	UNI_your
UNI_broke	UNI_enjoy	UNI_help	UNI_memori	UNI_processor	UNI_space	
UNI_budget	UNI_enough					
