# Supplementary material for: Confounds and overestimations in fake review detection: Experimentally controlling for product-ownership and data-origin
Source: PLoS One. 2022 Dec 7;17(12):e0277869. doi: 10.1371/journal.pone.0277869 (PMC9728858; doi:10.1371/journal.pone.0277869)
Supplement: S3 Table — (PDF) [file pone.0277869.s003.pdf]

Extra Trees classifier settings

n_estimators=100,<br>*,<br>criterion='gini',<br>max_depth=None,<br>min_samples_split=2,<br>min_samples_leaf=1,<br>min_weight_fraction_leaf=0.0,<br>max_features='auto',<br>max_leaf_nodes=None,<br>in_impurity_decrease=0.0,	bootstrap=False,<br>oob_score=False,<br>n_jobs=None,<br>random_state=319,<br>verbose=0,<br>warm_start=False,<br>class_weight=None,<br>ccp_alpha=0.0,<br>max_samples=None
