# Supplementary material for: Confounds and overestimations in fake review detection: Experimentally controlling for product-ownership and data-origin
Source: PLoS One. 2022 Dec 7;17(12):e0277869. doi: 10.1371/journal.pone.0277869 (PMC9728858; doi:10.1371/journal.pone.0277869)
Supplement: S4 Table — (PDF) [file pone.0277869.s004.pdf]

Other tested classifiers

Classifier
Random Forest
Decision Tree
MultinomialNB
GaussianNB
GradientBoosting
Logistic Regression
LinearSVC
