# Supplementary material for: Confounds and overestimations in fake review detection: Experimentally controlling for product-ownership and data-origin
Source: PLoS One. 2022 Dec 7;17(12):e0277869. doi: 10.1371/journal.pone.0277869 (PMC9728858; doi:10.1371/journal.pone.0277869)
Supplement: S5 Table — (PDF) [file pone.0277869.s005.pdf]

All classification performance metrics across all experiments

			Sentiment							
			Positive				Negative			
	**Analysis**	**Testing**	**Acc.**	**Precision**	**Recall**	**F1**	**Acc.**	**Precision**	**Recall**	**F1**
**Pure**	**1**	**Veracity**	60.26	60.54	60.23	60.04	69.87	70.24	69.87	69.76
	**2**	**Ownership**	63.41	63.87	63.41	63.07	58.61	58.65	58.62	58.54
	**3**	**Data-origin**	88.33	88.79	88.35	88.23	85.23	85.79	85.26	85.18
**Confounded**	**4**	**Veracity, Ownership**	66.19	66.43	66.17	66.06	74.17	74.41	74.17	74.09
	**5**	**Veracity, Data-origin**	86.94	87.35	86.94	86.91	84.44	84.62	84.47	84.43
	**6**	**Veracity, Ownership, Data-origin**	88.12	88.34	88.12	88.10	87.26	87.78	87.24	87.19
