# Supplementary material for: Confounds and overestimations in fake review detection: Experimentally controlling for product-ownership and data-origin
Source: PLoS One. 2022 Dec 7;17(12):e0277869. doi: 10.1371/journal.pone.0277869 (PMC9728858; doi:10.1371/journal.pone.0277869)
Supplement: S6 Table — (PDF) [file pone.0277869.s006.pdf]

Explanations of all feature names

Name	Meaning
**Part of Speech (POS) features**	
CD	cardinal digit
JJS	adjective, superlative (e.g., “biggest”)
**LIWC features**	
social	Social processes
WPS	Words/sentence
focuspresent	Time orientations: Present focus
focuspast	Time orientations: Past focus
money	Money
Tone	Emotional tone
conj	Conjunctions
see	Perceptual processes: See
i	Personal pronouns: 1st pers singular
Comma	Commas
Period	Periods
ppron	Personal pronouns
time	Relativity: Time
Exclam	Exclamation mark
percept	Perceptual processes
Authentic	Authentic
auxverb	Auxiliary verbs
article	Articles
function	Function Words
work	Personal concerns: Work
