# Supplementary material for: Confounds and overestimations in fake review detection: Experimentally controlling for product-ownership and data-origin
Source: PLoS One. 2022 Dec 7;17(12):e0277869. doi: 10.1371/journal.pone.0277869 (PMC9728858; doi:10.1371/journal.pone.0277869)
Supplement: Attachment — Submitted filename: Response to Reviewers.docx [file pone.0277869.s007.docx]

Dear Dr. Stefano Cresci,

We want to thank you and the two reviewers for the very helpful and encouraging comments on our paper. We integrated the suggestions into the current version, and responded to each comment in detail below.

Please find attached a version of the paper with track changes on (“Revised Manuscript with Track Changes”) and an unmarked version (“Manuscript”).

In the response letter, we included the reviewers’ comments in *italics*, our answers in **bold**, and the text changes in red font.

Kind regards from all authors,

Felix Soldner

(corresponding author: felix.soldner@ucl.ac.uk)

-----------------------------------------------------------------------------------------------------------------------------

Editor’s comment:

*In preparing this revision I suggest to carefully follow the thorough comments provided by the two reviewers. In particular, more effort should be devoted at critically analyzing the large body of related papers. In doing this, particular care should be given at emphasizing differences with respect to the 2020 paper by Fornaciari et al. Then, more details, explanations and experiments are also needed, as highlighted by both reviewers.*

**We followed each reviewers comment and provided specific feedback. In elaborating on the existing literature, which are highlighted in the individual responses seperately, we also included more details in how the 2020 paper by Fornaciari et al. differs to our work.**

[…] Both studies [30,31] hint at the problems of confounding factors in deception detection tasks. Although [31] uses a well-designed setup to test hypotheses, book reviews were not always about the same books between classes, introducing a potential content related confound. Similarly, the machine learning classifiers used were not always cross-validated with the same data type (i.e., the training and testing data were sourced from different data subsets), complicating the interpretation of the results. In contrast, in the current study, we match product types, hold the cross-validating procedure constant across all data subsets, and extend the analyses to positive and negative reviews.

Reviewers comments:

*Reviewer #1:*

*The manuscript considers possible confounding factors that may alter the results of a fake review classification task. Specifically, it considers two properties: the origin of the reviewer (e.g., social platform or crowdsourcing?) and whether or not the reviewer owns the product being reviewed.*

*In order to understand if the classifier is influenced by these two features, in addition to the veracity of the review, the authors build a review dataset that can be filtered according to three different aspects: the veracity of the review, the data origin of the reviewer, the ownership of the product by the reviewer.*

*The paper poses a very interesting problem, that is, how to build a suitable and non-biased dataset for a classification task*

*1.	A first point that puzzles me is that probably the dataset that the authors have built is also biased... certainly not because of the authors.First of all, as they themselves acknowledge, to consider Amazon reviews as truthful is to deny the very existence of the problem of fake reviews... And the authors are well aware that even certified purchases can be manipulated, as they point out in the discussion. Also, how can we be sure that Prolific reviewers aren’t lying? Unfortunately, that cannot be verified either, or at least I think so. I have tried registering on Prolific (thanks for introducing me to it, very interesting!), but they do not currently need annotators, so I do not know if they simply trust what an annotator says during registration, and if not, how do they verify the annotators’ supposed characteristics?*

*Anyway, I understand that this is really a big problem to solve. I think it could be useful to enrich the discussion to consider the problem of reliability of crowdsourcing platforms as well.*

**We acknowledge the problem of reliability in crowdsourcing approaches and integrated a section on that issue in the discussion section (under “Limitations”).**

5.4.2. Quality of Prolific reviews

We cannot be certain that Prolific participants who wrote the smartphones reviews were honest when instructed to be. However, previous research has shown that crowdsourcing platforms produce – in most cases – high-quality data and are better suited to the collection of large amounts of text data than other traditional collection methods, such as student samples [60,61]. Research also suggests that compared to other crowdsourcing platforms (e.g., Amazon Mechanical Turk, CloudResearch), Prolific seems to produce data of higher quality and with the most honest responses [62].

*2.	There is one assessment that seems to me a bit too exclusive, about data origin. Quoting from the text:*

*....`The first concerns the provenance of the data used. For example, deceptive reviews are often collected from participants recruited through crowdsourcing platforms, while truthful reviews are scraped from online platforms.’*

*This is true for the cited articles, the famous articles by Ott et al., but the literature also shows many examples of datasets built with reviewers with same data origin, e.g., with tags regarding veracity released by the same platforms (there are some work based on Yelp’s release of tags). Then, the training is done on that dataset, and then the trained classifier lis launched on another unknown dataset, where, maybe, the reviewer is of another origin (say, trained on Yelp and used on Amazon, for example..). I think the paper deserves a slightly more robust RW part. The following paper mentions different approaches in the Literature Review section. So maybe authors can take a cue:*

*Fazzolari et al.:*

*Experience: Improving Opinion Spam Detection by Cumulative Relative Frequency Distribution. ACM J. Data Inf. Qual. 13(1): 4:1-4:16 (2021)*

**We elaborated on other studies using “donated” data as well as scraped data for which review labels (truthful/fake) were obtained through hand crafted rules. We also included more references to studies re-using existing datasets.**

Introduction:

[...] Higher rates of performance are usually found in studies for which the review dataset [16–21] is constructed from several different sources, namely a crowdsourcing platform and an online review platform [13,14]. High classification performances are also found in studies using data scraped or donated from a single review platform, such as Yelp or Amazon [21–24]. Lower rates of performance are typically found in studies for which data is extracted from a single source and for which greater experimental control is exercised [10,25–27]. Why we can observe such strong differences of classification performances between studies, is unclear. However, such findings suggest that confounds associated with the construction of datasets may explain some of the variation in classification performance between studies and highlights the need for the exploration of such issues. [...]

1.1. Confounding factors:

[...] As such, ownership and review veracity (fake or genuine) will also be confounded.

Besides these two confounds, it is worth noting that some of the studies that have examined fake review detection have used scraped or donated data that does not have “ground truth ” labels [21–24,28,29]. That is, they have used data for reviews for which the veracity of the content is not known but is instead inferred through either hand-crafted rules (e.g., labeling a review as fake when multiple “elite” reviewers argue it is fake) or provided (non-transparently) by the platform’s owners (e.g., Yelp, Amazon). While utilizing such data to investigate how platforms filter reviews is helpful, studying the effects of deception without ground truth labels is problematic because any found class specific properties cannot be reliably attributed to the class label. Furthermore, any efforts to improve automated deception detection will be limited by algorithmically filtered data, because classification performances cannot exceed the preceding filter. Thus, ground truth labels are imperative for investigating deception detection in supervised approaches and such labels can be obtained through experimental study designs. However, previous studies collecting data experimentally [13,14] suffer from the confounds mentioned above and an altered study design is required.

*3.	We now come to performances. Unfortunately, I cannot interpret the results -or better, I cannot interpret the authors interpretation of the results :), so I need more information.*

*I quote:*

*….`leading researchers to conclude that a classifier can distinguish between truthful and fake reviews when, in reality, it is actually distinguishing between other characteristics of the data.’*

*Good, but the point is: do these additional features that the dataset has, which the authors call confounding factors, help or not to find fake reviews? If I understand correctly, yes! (Am I wrong, is there something I’m not clear on?). Because if I look at the performances, and read about overestimates, I don’t understand what this overestimation is). Can you give me an example where, in a certain scenario, confounding factors lead to classify fake reviews as true or vice versa? Is this the issue?*

**Thank you for this comment. We agree, that in this paper the confounding factors are supporting a distinction between truthful and fake reviews. We have clarified why leveraging the confounds for classification tasks is problematic. We included a sub-section “Practical implications” clarifying how the confounding factors in classification tasks will most likely lead to a model, which will not generalize well. We also made changes to the “aim of the paper” and “conclusion” section to highlight the distinction of classifications based solely on veracity and additional data characteristics.**

1.3. Aim of the paper:

Confounding variables have the potential to distort the findings of studies, leading researchers to conclude that a classifier can distinguish between truthful and deceptive reviews when, in reality, it is actually leveraging additional characteristics of the data, such as the way in which it was generated. [...]

[...] If *ownership* or *data-origin* do influence review content (we hypothesize that they do), reviews should be easier to differentiate when either of the two confounds is present in veracity classification, but reviews should be most easily classifiable if both confounds (*ownership, data-origin*) are present at the same time. Thus, our experiments allow us to assess how well a classifier can differentiate reviews based on veracity alone, and how much the confounds discussed above influence detection performances.

**5.4 Practical implications**

Previous studies that have examined the effects of supervised deception (veracity) detection suggest, that model performance does not easily transfer across domains, datasets or languages [49–52]. While domain specific language features (e.g., words specifically related to a product or service) probably contribute to the difficulties of model generalizability, other features related to the research setup or data collection procedures have also been candidate explanations for low model transferability [49]. The current study supports the idea, that the research design can have a strong effect on the classification performances in deception detection. Specifically, how the data is sourced (*data-origin*) seems to substantially effect classification performance. Consequently, models that are trained on data with confounds, such as in *data-origin* will most likely not generalize well, and will probably perform poorly when employed on data, which is sourced differently than the models’ training data. Thus, our findings indicate the importance of controlling for confounds in the training of classifiers.

6. Conclusion:

Through careful experimental control, we found that product *ownership* and *data-origin* do confound fake review detection. This may have resulted in the overestimation of model performances in detecting veracity in previous work. In particular, *data-origin* seems to boost classification performance, and this could easily be misattributed to the classification of *veracity* alone. Our findings suggest an overestimation of 24.89-46.23% when data is sourced from different platforms. Consequently, more effort and experimental control are necessary to create datasets when investigating complex concepts such as deception.

*Minor: the abstract is not very understandable, or rather, the first time I read it I understood very few.. Probably the authors can try rephrasing to introduce the reader more gently to the paper subject.*

**We revised the abstract to make it more understandable.**

**Abstract**

The popularity of online shopping is steadily increasing. At the same time, fake product reviews are published widely and have the potential to affect consumer purchasing behavior. In response, previous work has developed automated methods utilizing natural language processing approaches to detect fake product reviews. However, studies vary considerably in how well they succeed in detecting deceptive reviews, and the reasons for such differences are unclear. A contributing factor may be the multitude of strategies used to collect data, introducing potential confounds which affect detection performance. Two possible confounds are data-origin (i.e., the dataset is composed of more than one source) and product ownership (i.e., reviews written by individuals who own or do not own the reviewed product). In the present study, we investigate the effect of both confounds for fake review detection. Using an experimental design, we manipulate data-origin, product ownership, review polarity, and veracity. Supervised learning analysis suggests that review veracity (60.26 - 69.87%) is somewhat detectable but reviews additionally confounded with product-ownership (66.19 - 74.17%), or with data-origin (84.44 - 86.94%) are easier to classify. Review veracity is most easily classified if confounded with product-ownership and data-origin combined (87.78 - 88.12%). These findings are moderated by review polarity. Overall, our findings suggest that detection accuracy may have been overestimated in previous studies, provide possible explanations as to why, and indicate how future studies might be designed to provide less biased estimates of detection accuracy.

*In conclusion, I believe that it should be better clarified*

*a) if and how much bias could have been introduced while building the dataset.*

**See changes presented above (point 1)**

*b) why the authors talk about `confounding’ factors, do these factors help or not to find fake reviews?*

**See changes presented above (point 3)**

*c) in my opinion, the literature review on fake review detection should be expanded, with emphasis on datasets building.*

**See changes presented above (point 2)**

*Reviewer #2: The paper focuses on an intriguing question that the literature systematically underestimates. It also reads well and smoothly up to the end, even considering the quite intertwined set of experiments.*

*The results appear to be sound and supported by the plots. However, as reference 17 shows, the results are not completely unexpected. Moreover, there are several issues that the authors should consider addressing to improve the paper.*

*Firstly, the model used in the experiments is unclear and unexplained (maybe overlooked). Since the literature is flourishing with models of classifiers that excel (as often declared by authors) in detecting fake reviews, the authors could have explained their choice not to adopt available classifiers and build their own classifier.*

**We moved information about the classifier into the same paragraph (below the section “Supervised learning analysis”) and provided a list of the classifiers’ settings in the appendix. We also included a paragraph explaining our choice to train and test our own classifier.**

3. Supervised learning analysis

[...] Lastly, during feature preprocessing, features with a variance of 0 in each class (e.g., truthful-positive-owners, deceptive-positive-owners) were excluded to avoid any non-content related features (e.g., Amazon-specific website signs or words, such as “verified purchased”) affecting the results. Appendix B provides the list of features, which were present across all analyses. In total, we removed 69,927 (99.92%) bigrams, 6,520 (94.31%) unigrams, 13 (37.14%) POS features (WRB, WP$, WP, VBG, UH, TO, RBS, PRP, POS, PDT, EX, '', $), and 4 (4.3%) LIWC features (we, sexual, filler, female).

Instead of using a pre-trained model from other published work, we decided to train and test our own classifier. So doing meant that we could ensure that all observed changes in classification performance could be attributed to our experimental manipulations as opposed to other factors (e.g., domain differences or class imbalances in the training data of other models). We tested several different classifiers, but the “Extra Trees” classifier [44] showed the best performance in most scenarios and is, therefore, reported throughout all analyses (see Appendix C for the classifier settings and Appendix D for a list of other tested classifiers). All classification models were implemented in python with the “scikit-learn” package [45]. No hyperparameter changes were made.

*Similarly, the details of the used classifier are pretty limited (a Section with a total of 11 lines): the reader would expect at least the list of the final employed features and some additional elements to better figure out the mechanism chosen to process the dataset. Considering that the whole paper is centered on the effects of confounds on classifier performances, the reader should expect better awareness and care of such details. While the focus on the data is crucial, the reliability and quality of the classifier building are also critical and should not be neglected.*

**We provided a full list of the final feature set in Appendix B and the details of the classifier settings in Appendix C.**

**Appendix B: All features used in the classification experiments**

| Part of speech | | | | | | | | | | | | | | | | | | | | | |
| --- | --- | --- | --- | --- | --- | --- | --- | --- | --- | --- | --- | --- | --- | --- | --- | --- | --- | --- | --- | --- | --- |
| POS_CC  POS_CD  POS_DT | POS_FW  POS_IN  POS_JJ | | | | POS_JJR  POS_JJS  POS_MD | | POS_NN  POS_NNP  POS_NNS | | | | POS_PRP$  POS_RB  POS_RBR | | | | POS_RP  POS_VB  POS_VBD | | POS_VBN  POS_VBP  POS_VBZ | | | | POS_WDT |
| LIWC | | | | | | | | | | | | | | | | | | | | | |
| LIWC_Analytic  LIWC_Clout  LIWC_Authentic  LIWC_Tone  LIWC_WPS  LIWC_Sixltr  LIWC_Dic  LIWC_function  LIWC_pronoun  LIWC_ppron  LIWC_i  LIWC_you  LIWC_shehe  LIWC_they  LIWC_ipron  LIWC_article  LIWC_prep  LIWC_auxverb | | | | LIWC_adverb  LIWC_conj  LIWC_negate  LIWC_verb  LIWC_adj  LIWC_compare  LIWC_interrog  LIWC_number  LIWC_quant  LIWC_affect  LIWC_posemo  LIWC_negemo  LIWC_anx  LIWC_anger  LIWC_sad  LIWC_social  LIWC_family  LIWC_friend | | | | | LIWC_male  LIWC_cogproc  LIWC_insight  LIWC_cause  LIWC_discrep  LIWC_tentat  LIWC_certain  LIWC_differ  LIWC_percept  LIWC_see  LIWC_hear  LIWC_feel  LIWC_bio  LIWC_body  LIWC_health  LIWC_ingest  LIWC_drives  LIWC_affiliation | | | | | LIWC_achieve  LIWC_power  LIWC_reward  LIWC_risk  LIWC_focuspast  LIWC_focuspresent  LIWC_focusfuture  LIWC_relativ  LIWC_motion  LIWC_space  LIWC_time  LIWC_work  LIWC_leisure  LIWC_home  LIWC_money  LIWC_relig  LIWC_death  LIWC_informal | | | | | LIWC_swear  LIWC_netspeak  LIWC_assent  LIWC_nonflu  LIWC_AllPunc  LIWC_Period  LIWC_Comma  LIWC_Colon  LIWC_SemiC  LIWC_QMark  LIWC_Exclam  LIWC_Dash  LIWC_Quote  LIWC_Apostro  LIWC_Parenth  LIWC_OtherP | | |
| Bigrams | | | | | | | | | | | | | | | | | | | | | |
| BI_also_batteri  BI_android_phone  BI_batteri_life  BI_bought_phone  BI_camera_good  BI_camera_phone  BI_camera_qualiti  BI_cheap_phone  BI_dont_know  BI_dont_want  BI_even_though | | | BI_everi_day  BI_front_camera  BI_good_phone  BI_good_price  BI_great_phone  BI_intern_storag  BI_iphon_7  BI_ive_ever  BI_ive_phone  BI_last_day  BI_last_long | | | | | BI_like_phone  BI_look_good  BI_much_better  BI_new_phone  BI_oper_system  BI_phone_batteri  BI_phone_camera  BI_phone_ever  BI_phone_everyth  BI_phone_good  BI_phone_ive | | | | | BI_phone_much  BI_phone_price  BI_phone_realli  BI_phone_screen  BI_phone_work  BI_phone_would  BI_pretti_good  BI_qualiti_phone  BI_realli_good  BI_recommend_phone | | | | | BI_samsung_galaxi  BI_sd_card  BI_smart_phone  BI_sound_qualiti  BI_take_photo  BI_use_phone  BI_want_phone  BI_work_well  BI_would_recommend | | | |
| Unigrams | | | | | | | | | | | | | | | | | | | | | |
| UNI_1  UNI_10  UNI_2  UNI_2020  UNI_3  UNI_4  UNI_5  UNI_6  UNI_7  UNI_8  UNI_abl  UNI_absolut  UNI_access  UNI_actual  UNI_addit  UNI_afford  UNI_ago  UNI_allow  UNI_almost  UNI_alreadi  UNI_also  UNI_although  UNI_alway  UNI_amaz  UNI_amount  UNI_android  UNI_annoy  UNI_anoth  UNI_anyon  UNI_anyth  UNI_app  UNI_appl  UNI_applic  UNI_arent  UNI_around  UNI_ask  UNI_avail  UNI_away  UNI_back  UNI_bad  UNI_basic  UNI_bateri  UNI_batteri  UNI_beauti  UNI_believ  UNI_besid  UNI_best  UNI_better  UNI_big  UNI_bigger  UNI_bit  UNI_bought  UNI_brand  UNI_bright  UNI_bring  UNI_broke  UNI_budget | | UNI_buy  UNI_call  UNI_came  UNI_camera  UNI_cant  UNI_card  UNI_case  UNI_caus  UNI_cellphon  UNI_chang  UNI_charg  UNI_cheap  UNI_cheaper  UNI_choic  UNI_clear  UNI_color  UNI_come  UNI_compani  UNI_compar  UNI_complet  UNI_condit  UNI_connect  UNI_consid  UNI_cool  UNI_cost  UNI_could  UNI_couldnt  UNI_coupl  UNI_cover  UNI_crash  UNI_current  UNI_custom  UNI_daili  UNI_damag  UNI_data  UNI_day  UNI_deal  UNI_decent  UNI_decid  UNI_definit  UNI_design  UNI_devic  UNI_didnt  UNI_differ  UNI_disappoint  UNI_display  UNI_doesnt  UNI_dont  UNI_download  UNI_drop  UNI_due  UNI_easi  UNI_easili  UNI_els  UNI_end  UNI_enjoy  UNI_enough | | | | UNI_especi  UNI_etc  UNI_even  UNI_ever  UNI_everi  UNI_everyday  UNI_everyth  UNI_excel  UNI_except  UNI_expect  UNI_expens  UNI_experi  UNI_extra  UNI_extrem  UNI_fact  UNI_fail  UNI_fall  UNI_far  UNI_fast  UNI_faster  UNI_featur  UNI_feel  UNI_find  UNI_fine  UNI_fingerprint  UNI_first  UNI_fit  UNI_flagship  UNI_found  UNI_freez  UNI_friend  UNI_front  UNI_full  UNI_function  UNI_galaxi  UNI_game  UNI_gave  UNI_gb  UNI_gener  UNI_get  UNI_give  UNI_given  UNI_go  UNI_goe  UNI_good  UNI_googl  UNI_got  UNI_great  UNI_half  UNI_hand  UNI_handl  UNI_happi  UNI_hard  UNI_hardwar  UNI_heavi  UNI_help | | | | UNI_high  UNI_hit  UNI_home  UNI_honestli  UNI_hour  UNI_howev  UNI_huawei  UNI_huge  UNI_id  UNI_im  UNI_imag  UNI_import  UNI_impress  UNI_includ  UNI_instal  UNI_intern  UNI_internet  UNI_iphon  UNI_isnt  UNI_issu  UNI_ive  UNI_job  UNI_keep  UNI_know  UNI_lack  UNI_lag  UNI_larg  UNI_last  UNI_least  UNI_less  UNI_let  UNI_lg  UNI_life  UNI_light  UNI_like  UNI_line  UNI_littl  UNI_live  UNI_load  UNI_long  UNI_longer  UNI_look  UNI_lose  UNI_lot  UNI_love  UNI_low  UNI_lower  UNI_made  UNI_main  UNI_make  UNI_mani  UNI_market  UNI_may  UNI_mayb  UNI_meanUNI_memori | | UNI_mention  UNI_might  UNI_mine  UNI_mobil  UNI_model  UNI_money  UNI_month  UNI_much  UNI_multipl  UNI_music  UNI_must  UNI_need  UNI_never  UNI_new  UNI_next  UNI_nice  UNI_normal  UNI_noth  UNI_notic  UNI_offer  UNI_often  UNI_ok  UNI_okay  UNI_old  UNI_one  UNI_open  UNI_oper  UNI_option  UNI_os  UNI_other  UNI_overal  UNI_own  UNI_pay  UNI_peopl  UNI_perform  UNI_person  UNI_phone  UNI_photo  UNI_pick  UNI_pictur  UNI_piec  UNI_pixel  UNI_play  UNI_plu  UNI_pocket  UNI_point  UNI_poor  UNI_power  UNI_present  UNI_pretti  UNI_previou  UNI_price  UNI_pro  UNI_probabl  UNI_problem  UNI_processor | | | | UNI_product  UNI_purchas  UNI_put  UNI_qualiti  UNI_quickli  UNI_quit  UNI_ram  UNI_rang  UNI_rate  UNI_rather  UNI_read  UNI_real  UNI_realli  UNI_reason  UNI_recent  UNI_recommend  UNI_record  UNI_releas  UNI_reliabl  UNI_resist  UNI_resolut  UNI_respons  UNI_review  UNI_right  UNI_run  UNI_samsung  UNI_satisfi  UNI_say  UNI_scratch  UNI_screen  UNI_sd  UNI_second  UNI_see  UNI_seem  UNI_servic  UNI_set  UNI_sever  UNI_short  UNI_show  UNI_simpl  UNI_simpli  UNI_sinc  UNI_singl  UNI_size  UNI_slightli  UNI_slow  UNI_small  UNI_smart  UNI_smartphon  UNI_softwar  UNI_someon  UNI_someth  UNI_sometim  UNI_soon  UNI_sound  UNI_space | | | | UNI_spec  UNI_specif  UNI_speed  UNI_spend  UNI_star  UNI_start  UNI_still  UNI_storag  UNI_store  UNI_super  UNI_support  UNI_suppos  UNI_sure  UNI_system  UNI_take  UNI_thank  UNI_that  UNI_thing  UNI_think  UNI_though  UNI_time  UNI_took  UNI_top  UNI_total  UNI_touch  UNI_tri  UNI_turn  UNI_two  UNI_updat  UNI_upgrad  UNI_usag  UNI_use  UNI_user  UNI_valu  UNI_version  UNI_video  UNI_want  UNI_wasnt  UNI_watch  UNI_way  UNI_week  UNI_well  UNI_whole  UNI_within  UNI_without  UNI_wont  UNI_work  UNI_worth  UNI_would  UNI_wouldnt  UNI_wrong  UNI_x  UNI_xiaomi  UNI_year  UNI_your | |

**Table 6** List of all features

**Appendix C: Extra Trees classifier settings**

| n_estimators=100,  *,  criterion='gini',  max_depth=None, min_samples_split=2, min_samples_leaf=1, min_weight_fraction_leaf=0.0, max_features='auto', max_leaf_nodes=None,  in_impurity_decrease=0.0, | bootstrap=False,  oob_score=False,  n_jobs=None,  random_state=319,  verbose=0,  warm_start=False, class_weight=None,  ccp_alpha=0.0,  max_samples=None |
| --- | --- |

**Table 7** Extra Trees classifier settings

*Finally, there are also some disregard, maybe naive, observations about why the obtained results are strikingly evident when comparing crowdsourced reviews with Amazon’s ones. In Amazon’s reviews, it is somewhat expected to encounter considerations to elements out of the reviewed product itself, namely references to the shipping time, delivery status, customer service experiences, returns, refunds, refurbished products, and similar. While the authors in Section 4 refer to some form of avoidance of non-content related features, there is no reference to such (expected?) bias in the text of Amazon’s reviews. A similar statement could be made when dealing with the owner and not-owner experiment: reference to relative first-person pronouns and adjectives could be expected to be higher in the owner group. This and the above observations could appear to be easy biases that the authors could further explore to explain their thesis better.*

**Agreed. To understand how reviews differ linguistically we performed Bayesian hypothesis testing on the features present in each classification task. Thus, we included the following analyses section as well as the discussion section:**

4.2. Linguistic properties in pure and confounded classification experiments

The linguistic properties of all analyses were examined using Bayesian hypothesis testing [46–48]. The aim was to investigate which linguistic features drive each classification. To do this, we inspected the top 5 highest Bayes Factors (BF_10_) and reported features with scores of 10 or greater. A BF_10_ indicates the likelihood of the data if there was a difference of occurrences in the feature between the compared classes (alternative hypothesis) relative to the null hypothesis (no difference). A BF_10_ of 1 represents an equal likelihood of the null- and alternative hypothesis. Each reported feature name is tagged with one of the following indications: “POS” (part of speech), “LIWC” (Linguistic Inquiry and Word Count), “UNI” (unigram), “BI” (bigrams), to categorize its feature type. Table 3 shows the top 5 linguistic features for the pure classification experiments, while table 4 shows the top 5 features for the classification experiments in which we introduced confounds.

|  | | **Experiment** | | |
| --- | --- | --- | --- | --- |
| **Testing** | | **Veracity** | **Ownership** | **Data-origin** |
| **Sentiment** | **Positive** | LIWC_social (-) | POS_CD (-) | LIWC_Comma (+) |
|  |  | POS_CD (+) | LIWC_see (-) | UNI_smartphon (+) |
|  |  | UNI_phone (-) | UNI_im (-) | UNI_camera (+) |
|  |  | UNI_iphon (+) | LIWC_WPS (-) | LIWC_social (-) |
|  |  | LIWC_WPS (+) | LIWC_i (-) | UNI_best (+) |
|  | **Negative** | LIWC_focuspresent (-) | UNI_samsung (+) | UNI_smartphon (+) |
|  |  | LIWC_focuspast (+) |  | LIWC_Comma (+) |
|  |  | LIWC_money (-) |  | UNI_slow (+) |
|  |  | LIWC_Tone (+) |  | LIWC_Period (+) |
|  |  | LIWC_conj (+) |  | LIWC_ppron (-) |

**Table 3** Top 5 feature differences by BF_10_ for all pure classifications; BF_10_>11 for each feature; (+) = feature appears more often in truthful reviews, in reviews by smartphone non-owners, or in Prolific reviews; (-) = feature appears more often in deceptive reviews, in reviews by smartphone owners, or in Amazon reviews; See Appendix F for all feature explanations.

|  | | **Experiment** | | |
| --- | --- | --- | --- | --- |
| **Testing** | | **Veracity,**  **Ownership** | **Veracity,**  **Data-origin** | **Veracity,**  **Ownership,**  **Data-origin** |
| **Sentiment** | **Positive** | UNI_year (+) | UNI_smartphon (-) | UNI_camera (-) |
|  |  | LIWC_time (+) | UNI_camera (-) | UNI_smartphon (-) |
|  |  | LIWC_Exclam (-) | LIWC_Authentic (+) | LIWC_article (-) |
|  |  | LIWC_percept (-) | LIWC_auxverb (-) | LIWC_function (-) |
|  |  | UNI_still (+) | POS_JJS (-) | UNI_photo (-) |
|  | **Negative** | LIWC_focuspast (+) | LIWC_focuspast (+) | UNI_smartphon (-) |
|  |  | LIWC_money (-) | LIWC_Authentic (+) | LIWC_Comma (-) |
|  |  | LIWC_focuspresent (-) | UNI_qualiti (-) | LIWC_work (+) |
|  |  | UNI_buy (-) | UNI_bad (-) | UNI_slow (-) |
|  |  | LIWC_Exclam (-) | LIWC_social (+) | UNI_bad (-) |

**Table 4** Top 5 feature differences by Bayes Factor_10_ for all confounded classifications; BF_10_>188 for each feature; (+) = feature appears more often in truthful reviews; (-) = feature appears more often in deceptive reviews; See Appendix F for all feature explanations*.*

**(In Appendix F)**

| **Name** | **Meaning** |
| --- | --- |
| **Part of Speech (POS) features** | |
| CD | cardinal digit |
| JJS | adjective, superlative (e.g., “biggest”) |
| **LIWC features** | |
| social | Social processes |
| WPS | Words/sentence |
| focuspresent | Time orientations: Present focus |
| focuspast | Time orientations: Past focus |
| money | Money |
| Tone | Emotional tone |
| conj | Conjunctions |
| see | Perceptual processes: See |
| i | Personal pronouns: 1st pers singular |
| Comma | Commas |
| Period | Periods |
| ppron | Personal pronouns |
| time | Relativity: Time |
| Exclam | Exclamation mark |
| percept | Perceptual processes |
| Authentic | Authentic |
| auxverb | Auxiliary verbs |
| article | Articles |
| function | Function Words |
| work | Personal concerns: Work |

**Table 10** Explanations of all feature names

**(In the Discussion section)**

**5.3. Linguistic properties**

Examining the top 5 features ranked by their Bayes Factor_10_ for each classification task provides insight into each class's text differences. As expected from the classification performance metrics, we observe that features are not consistent across sentiment nor between or across classes.

Both deceptive and truthful reviews highlight non-psychological or non-perceptual constructs (except LIWC_social). Given the increased usage of “phone” in deceptive positive reviews, individuals writing such reviews might reiterate what review type they are writing. Similarly, truthful review writers seem to highlight that their product originates from the brand Apple. Truthful reviews also seem to focus on the past, which might support the idea of highlighting past-owned phones. Thus, truthful negative reviews might exhibit a stronger emphasis on the ownership of the phone and when it was owned. In turn, deceptive negative reviews seem to focus more on the present and money, suggesting that the phone price might be over-emphasized in such reviews. For differences in *ownership*, we observe fewer differences, but smartphone owners seem to express their experience more in personal terms than do non-owners. However, this was only the case for positive reviews. Prolific reviews seem to follow a more factual, syntactical, and sentiment-specific style, suggesting a stronger emphasis on the product. In contrast, Amazon reviews seem to include more social processes (LIWC_social) and personal pronounces (LIWC_ppron), which could be attributed to an increased focus on services (delivery, refunds, customer support, etc.). However, the increased usage of the words “best” and “slow” in Prolific reviews could serve a similar function.

Lastly, an examination of the linguistic properties of the confounded classification experiments shows a mixed picture of classification features that appeared in the initial experiment (i.e., when no confounds were introduced) and some new ones. For example, feature differences for the classification of *veracity* and *ownership* show a new set of features for positive reviews. For the negative reviews, however, the features identified were those previously seen in the pure *veracity* classification. Since almost no differences were present for the *ownership* condition features for negative reviews, such an effect seemed expected. Interestingly, when *veracity* is confounded with *data-origin*, or with *ownership*, and *data-origin* combined, we see recurring features from the purely *data-origin* classification, which is strongest when both confounds are present. Thus, *data-origin* seems to have a strong linguistic impact, which is reflected in strong classification performance.

*Minor typos:*

*-the value in the last column of Amazon owners row of table 2 appears to be a copy-n-paste typo.*

**Thank you for noticing that error, we corrected table 2.**

|  | **Truthful** | | **Deceptive** | | **Total** |
| --- | --- | --- | --- | --- | --- |
| **Review type** | Pos | Neg | Pos | Neg |  |
| Prolific owners | 384 | 327 | 302 | 348 | **1361** |
| Prolific non-owners | - | - | 352 | 371 | **723** |
| Amazon owners | 1038 | 1046 | - | - | **2084** |
| **Total** | **1422** | **1373** | **654** | **719** | **4168** |

**Table 2** Overview of all filtered reviews

*-the double rounded arrow of Figure 1 is misleading: what does it mean? Maybe another label could help.*

**The double rounded arrow in Figure 1 was intended to illustrate that the different conditions are counterbalanced and do not always follow the shown order. Since the counterbalancing is mentioned in the text, we changed the double rounded arrows to a single downward arrow, to make Figure 1 clearer.**
