# Supplementary material for: Confounds and overestimations in fake review detection: Experimentally controlling for product-ownership and data-origin
Source: PLoS One. 2022 Dec 7;17(12):e0277869. doi: 10.1371/journal.pone.0277869 (PMC9728858; doi:10.1371/journal.pone.0277869)
Supplement: Attachment — Submitted filename: Response to Reviewers.docx [file pone.0277869.s008.docx]

Dear Dr. Stefano Cresci,

We want to thank the reviewer for the helpful comments on our paper. We integrated the suggestions into the current version and responded to each comment in detail below.

Please find attached a version of the paper with track changes (“Revised Manuscript with Track Changes”) and an unmarked version (“Manuscript”).

In the response letter, we included the reviewers’ comments in *italics*, our answers in **bold**, and the text changes in red font.

Please let us know if you require anything further from us at this stage.

Kind regards from all authors,

Felix Soldner

(corresponding author: felix.soldner@ucl.ac.uk)

-----------------------------------------------------------------------------------------------------------------------------

Reviewers’ comments:

*Reviewer #1:*

*1) In articles that exploit data from the platforms themselves, like Yelp's, I am not 100% sure that the data is actually donated by the platform...I rather believe that the content of the site was pulled down by crawlers or scrapers, and that a part of reviews was defined as false because of hand crafted rules, like user alerts, while a part is the so called `filtered reviews', the ones that Yelp decided not to make public, because fake according to its algorithm, but that by scraping could be found...
thus, I'd ask the authors to possibly check the passage inserted in Section 1.1, to make sure the claims are correct.*

**Thank you for drawing our attention to that mistake. We corrected the subsection accordingly:**

[…]

Besides these two confounds, it is worth noting that some of the studies that have examined fake review detection have used scraped ~~or donated~~ data that does not have “ground truth ” labels [21–24,28,29]. That is, they have used data for reviews for which the veracity of the content is not known but is instead inferred through either hand-crafted rules (e.g., labeling a review as fake when multiple “elite” reviewers argue it is fake) or inferred by the platforms own filtering system, which is non-transparent (e.g., Yelp).

[…]

*2) As mentioned, I am very pleased that abstract, intro, conclusions... are now smoother and the article goal clearer.
Many times, however, the concept of confounding factors is repeated, and how they may have a bearing on the classifier's outcome. For my personal taste, I would avoid too much repetition of the concept. I leave it up to the editor to decide on this aspect, which is more about style.*

**Thank you for mentioning that aspect of the paper. Although the confounding factors are described several times, the context of these explanations changes slightly (e.g., within related work or theoretical and practical implications) to support a better understanding. Since the confounds are a central aspect of the paper, which also inform the experimental design setup, their understanding is essential. Thus, we believe that their repeated explanation would benefit readers that are not as familiar with the matter.**
